# Treatment utilization and treatment barriers in individuals with body dysmorphic disorder

**DOI:** 10.1186/s12888-020-02489-0

**Published:** 2020-02-18

**Authors:** Johanna Schulte, Claudia Schulz, Sabine Wilhelm, Ulrike Buhlmann

**Affiliations:** 1 Institute of Psychology, University of Münster, Fliednerstraße 21, 48149 Münster, Germany; 2 Present address: AMEOS Hospital Osnabrück, Knollstraße 31, Osnabrück, Germany; 3 Department of Psychiatry, Massachusetts General Hospital/Harvard Medical School, Simches Research Building, 185 Cambridge Street, Boston, MA 02114 USA

**Keywords:** Appearance concerns, Body dysmorphic disorder, Body image, Cosmetic surgery, Help-seeking, Insight, Mental health care, Self-test, Treatment barriers, Treatment utilization

## Abstract

**Background:**

Although effective treatments are available, most individuals with body dysmorphic disorder (BDD) do not receive an appropriate diagnosis or treatment. We aimed to examine treatment utilization and barriers to treatment, and to identify associated socio-demographic and clinical characteristics.

**Methods:**

German individuals completed an online self-report survey of appearance concerns. A sample of *N* = 429 individuals met criteria for BDD. We examined the frequency of treatment utilization and barriers, analyzed comparisons between treated and untreated individuals and assessed the relationships of socio-demographic and clinical features with mental health treatment utilization and treatment barriers, respectively.

**Results:**

Only 15.2% of the individuals with BDD had been diagnosed with BDD, and lifetime rates of mental health treatment were low (39.9%). Individuals endorsed multiple barriers to mental health treatment, especially shame, low perceived need and a preference for cosmetic and medical treatments. Associated features were identified, including age, a BDD diagnosis, body dysmorphic symptom severity, a likely major depressive disorder, prior cosmetic surgery, and insight.

**Conclusions:**

The results of this largest study to date highlight that BDD is still underrecognized and undertreated even in a country with extensive mental health care and few financial barriers. We discuss modifiable factors and strategies to foster awareness of BDD in sufferers and professionals to improve treatment dissemination and to reduce treatment barriers.

## Background

Body dysmorphic disorder (BDD) is a mental disorder characterized by an extensive preoccupation with perceived appearance flaws. BDD sufferers often spend many hours a day thinking about their appearance and frequently engage in rituals to improve or hide the body areas of concern. They are also often impaired with regard to social and occupational or academic functioning, leading to poor quality of life [1] or being housebound and socially isolated [2, 3]. Suicide attempts in adults with BDD are 2.6 fold increased in comparison to mentally healthy controls and patients with other disorders such as obsessive-compulsive disorder (OCD) and anorexia nervosa [4]. Psychological treatment with cognitive-behavioral therapy (CBT) and psychopharmacological treatment with selective serotonin-reuptake inhibitors (SSRIs) are the gold standard, empirically supported treatments for BDD [5–8].

 Despite being relatively common (i.e. weighted point prevalence rates of 1.9% in the community, 7.4% in inpatients and 5.8% in outpatients [9]), awareness for BDD is still lower than for other, comparably less common mental disorders like anorexia nervosa or schizophrenia. To some extent, this may be explained by an adverse pattern observed in prior studies. Veale et al. [10] reported that of those individuals identified as having BDD in an inpatient ward, none presented their appearance concerns during intake until specifically asked about them and none had been diagnosed with BDD by the clinicians who referred them to inpatient care. Thus, BDD tends to be underdiagnosed or mistaken for other disorders like depression or anxiety disorders [9, 11–15]. Consequently, BDD is frequently untreated or inadequately treated. Buhlmann [16], for example, found in an internationally recruited online-sample of 172 individuals with BDD that only 18.6% were currently receiving psychopharmacological medications and 19.8% were engaged in psychotherapy, of whom half were in CBT. In a similar study, Marques et al. [17] reported that out of 401 individuals with BDD, only 17.4% had received CBT, and 34.4% had been prescribed SSRIs. As BDD has a chronic course with low rates of spontaneous remission [18], untreated BDD not only causes a persistent psychological burden for BDD sufferers, but often also causes high costs for the health care system as well as BDD sufferers, e.g. due to disability and ineffective, nonspecialized treatments and expensive cosmetic treatments.

Barriers to treatment utilization in BDD may manifest in three areas. First, feelings of shame are strongly associated with BDD [19] and the stigma associated with suffering from a mental illness might deter individuals with BDD from disclosing their concerns [10, 16, 17]. Second, psychological or psychiatric treatments are often perceived to be ineffective [10, 16, 17]. Many BDD sufferers are strongly convinced of the real existence and visibility of their perceived flaws [20] and thus show poor insight into a psychological understanding of their symptoms or even delusionality towards the physical understanding, respectively. Therefore, many of them choose cosmetic treatments [9] over psychological interventions. Cosmetic procedures, however, are generally associated with poor outcomes for individuals with BDD and do not reduce BDD symptomatology [21]. Third, logistic and financial barriers, especially concerns about insurance coverage and a lack of knowledge about appropriately trained treatment providers, might prevent individuals from help-seeking [16, 17].

To date, there is only limited research on treatment utilization in BDD. Buhlmann [16] found that individuals with BDD who had been diagnosed with BDD by a professional reported more severe appearance concerns than those without an assigned BDD diagnosis. There were no differences with respect to age, educational level, and depressive symptoms. In line with this, Phillips et al. [22] compared currently treated and untreated individuals with diagnosed BDD and found that those in treatment had a higher lifetime comorbidity for any mood disorder, OCD and eating disorders. Moreover, currently treated individuals reported more body areas of concerns and greater functional impairments. The authors discussed a treatment-seeking bias by those with greater symptom severity and impairment. Further, currently treated individuals had better insight, which is likely to represent a positive effect of treatment or a sample bias as delusional individuals might be less engaged in treatment [22, 23]. In that study, however, even in the currently untreated subgroup, 86.4% had received treatment in the past and the results cannot be generalized to completely treatment-naïve individuals with BDD.

In sum, previous studies either used samples with high rates of treatment experience or did not analyze predictors of treatment seeking in detail. A deeper understanding of barriers and determinants of treatment is crucial for a targeted reduction of treatment barriers and a broader dissemination of treatment, especially for those individuals who are still unsure whether to initiate contact with a professional.

In this study, we analyzed the responses of participants to an online survey for appearance concerns. In an anonymous online setting, prominent treatment barriers related to personal contact interfere less with participation, e.g. housebound BDD sufferers are not required to leave the house and reporting sensitive symptoms associated with shame is facilitated [24]. Thus, we anticipated a representative sample of BDD sufferers with differing stages of motivation for treatment, i.e. including a substantial number of untreated individuals. We had the following aims: First, we examined treatment utilization and treatment barriers in individuals with BDD. Secondly, we aimed to identify socio-demographic and clinical characteristics that are associated with mental health treatment seeking and that impact perceived treatment barriers.

## Methods

### Procedure

We developed an online survey for appearance concerns, which included programmed feedback on the extent of appearance concerns and information about BDD and treatment options. This self-test was available on a website of a specialized psychotherapeutic outpatient clinic for BDD at the University of Münster, Germany. There was no active recruitment for the survey; however, the BDD program itself was promoted regularly in local newspapers and via flyers. The anonymous internet survey was designed on “Unipark” [25]. On the first two pages, participants were informed about the voluntary and confidential nature of the survey and were asked for their informed consent before continuing. Participants then filled out several questionnaires. After completing the survey, participants had to reconfirm informed consent for data use. Mean duration was 20 min 43 s (SD = 10 min 43 s). The participants received automated feedback regarding their extent of appearance concerns (e.g. “Your scores are heightened. Your answers indicate that you are very distressed by your appearance concerns and often preoccupied with your perceived appearance defects.”), and it was clarified that the feedback would not include a diagnosis of BDD or another mental disorder. The feedback further included general information on diagnostic criteria for BDD, common differential diagnoses, and BDD treatment as well as contact information for treatment sites in Germany. If a participant reported any suicidal thoughts (Patient’s Health Questionnaire depression scale Item 9), a crisis hotline number was provided.

### Participants

Between February 2016 and May 2018, 6652 individuals entered the self-test. Of those entering, *n* = 4696 discontinued on the first three pages, *n* = 545 discontinued in the further course, and *n* = 3 were excluded due to technical problems. Furthermore, those meeting exclusion criteria (no consent for data use (*n* = 340), repeated participation (*n* = 30), years of age under 18 (*n* = 88), not living in Germany (*n* = 126), not meeting diagnostic criteria for BDD according to the 5th edition of the Diagnostic and Statistical Manual of Mental Disorders (DSM-5, [26]; *n* = 395), were discarded, resulting in a final sample of 429 German-speaking participants with BDD.

### Measures

Participants reported gender, age, highest educational degree, occupational and marital status as demographic characteristics (see Table 1).

**Table 1:** Demographic characteristics (*n* = 429)

Demographic variables	*%*	*(n)*
Highest educational degree		
None	0.7	(3)
10 years of school	28.7	(123)
11 to 13 years of school	36.1	(155)
University or college	34.3	(147)
Other	0.2	(1)
Occupational status ^a^		
Student	39.6	(170)
Full-time	29.6	(127)
Part-time	21.2	(91)
Unemployed	6.3	(27)
Housewife/parental leave	5.6	(24)
On disability	2.6	(11)
Other	6.8	(29)
Marital status		
Single	49.4	(212)
Dating/cohabitating	35.0	(150)
Married	11.9	(51)
Separated/divorced	3.5	(15)
Widowed	0.2	(1)

Note. ^a^ Multiple choice item

The DSM-5 criteria for BDD were used as self-report items to screen for BDD [27]. Participants were asked to indicate whether they “fully agree”, or “do not agree at all” with each of the following items covering the DSM-5 criteria for BDD: appearance-related preoccupation (A), repetitive behaviors (B1), mental acts (B2), distress (C1), impairment (C2). In addition, participants were asked whether the appearance concerns are primarily weight related or not, as an affirmative answer could suggest the presence of an eating disorder (D). To qualify for BDD, participants needed to affirm criteria A, B1 and/or B2, C1 and/or C2 with “fully agree” and report no primary weight related concerns in criterion D. In an unpublished data set, the questionnaire showed a sensitivity of .76 and a specificity of 1 to differentiate 30 individuals with a BDD-diagnosis (according to the Structured Clinical Interview for DSM-IV [28]) from 30 mentally healthy controls.

To further screen for possible comorbid eating disorders, participants completed the SCOFF questionnaire [29, 30] by indicating “yes” or “no” on five items on disordered eating (e.g., “Do you worry you have lost control over how much you eat?”). The cut-off of two or more affirmed items has proven good diagnostic accuracy for the detection of eating disorders with pooled estimates of .80 for sensitivity and .93 for specificity in a meta-analysis of studies using different translations [31].

Body dysmorphic symptom severity in the past week was assessed with the 18-item Body Dysmorphic Symptoms Inventory (Fragebogen körperdysmorpher Symptome, FKS [32]). Further, the FKS includes a body area checklist (item 2) and items on insight (item 10 “conviction about appearance-related beliefs”), lifetime cosmetic surgery (item 16), and lifetime appearance-related suicide attempts (item 18) and cosmetic treatments. Items were judged on 4-point Likert scales (0 = “not at all or never” and 4 = “very strongly or more than 8 hours per day”) with a maximum sum score of 64 and a cut-off sum score of 14 to screen for BDD (sensitivity = .87, specificity = .93; Buhlmann et al., 2009). The internal consistency in our study was α = .80.

The Patient’s Health Questionnaire depression scale (PHQ-9) [33, 34] is a 9-item questionnaire assessing depression symptoms in the past 2 weeks on a 4-point Likert scale (0 = “not at all”, 1 = “several days”, 2 = “more than half the days”, 3 = “nearly every day”). It was used to screen for a likely major depressive disorder according to DSM-5 (sensitivity = .95 and specificity = .86) [35]. The internal consistency in our study was α = .87.

A treatment history questionnaire was presented to assess lifetime treatment utilization and other coping strategies, e.g. “Have you ever done anything in particular to address your appearance concerns?” including options for contacting mental health services, general health services and informal help. If applicable, participants also reported their lifetime diagnoses of mental disorders given by a health professional.

The participants were asked to indicate “yes” or “no” on the 20-item Barriers to Treatment Questionnaire (BTQ) [36] to assess barriers associated with non-treatment or delayed treatment help-seeking for appearance concerns on the subscales “logistic/financial barriers”, “stigma/shame/discrimination barriers” and “treatment satisfaction/perception barriers”. Specifically, we asked participants to answer with respect to seeking psychological or psychiatric treatment for their appearance concerns. We used a slightly modified version of the BTQ and added six additional items to the BTQ to include common BDD-specific treatment barriers found by Buhlmann [16]. We analyzed treatment barriers descriptively and used the total scale and single items, respectively. The internal consistency of the total scale including the additional items was acceptable (α = .70).

### Statistical analyses

SPSS version 24.0 was used for data analyses. We report descriptive analyses for sample characteristics as well as frequencies of treatment use and barriers to treatment. Since we were interested in the overall use of mental health services, we merged the lifetime use of psychotherapy, psychopharmacological medication and psychiatric inpatient care into a combined variable with individuals who used (vs. did not use) one or more of these mental health care services. We further dichotomized the answers to FKS item 16 on cosmetic surgery and FKS item 18 on suicide attempts to indicate if they did or did not apply at least once.  

To analyze the association of socio-demographic and clinical factors with mental health care utilization, we first compared treated and untreated individuals via t-tests (age, BDD symptom severity, insight in BDD, and number of treatment barriers) and Chi-square tests of independence (gender, current relationship status, BDD diagnosis, likely eating disorder, likely major depressive disorder, past appearance-related suicide attempts, and use of cosmetic surgery). The effect sizes Cohen’s *d* or Cramer’s *V* are reported. With respect to the highest educational degree, we report the results of Fisher’s exact test as the categories “other” and “none” did not meet the assumption of expected frequencies larger than five. To control the family-wise error rate in all multiple comparisons, we adjusted the *p*-values based on Bonferroni correction.

To estimate the relative effects of all socio-demographic and clinical predictors as well as the total number of treatment barriers on mental health treatment utilization, we used hierarchical logistic regression and report adjusted odds ratios. To examine the association of socio-demographic and clinical factors with treatment barriers, we computed a hierarchical multiple regression for the prediction of the total number of treatment barriers and report standardized beta values. Further hierarchical logistic regressions were analyzed with respect to selected individual barriers. We used a two-tailed α < .05 as level of significance in all analyses.

## Results

### Sample characteristics

The participants in the final sample were between 18 and 66 years old (*M* = 30.23, *SD* = 9.67). The majority (66.7%, *n* = 286) of the sample was female. See Table 1 for additional demographic details. The mean (*SD*) FKS score was 37.29 (8.37), indicating high BDD symptom severity. In addition, all but one of the participants met the cut-off on the FKS (range 12–57). The conviction about the appearance-related beliefs (FKS item 10) was moderate to strong (*M* = 3.27, *SD* = 0.87), indicating poor insight. Almost half of the participants met symptoms for a current major depressive disorder (53.4%, *n* = 229), and 27.5% (*n* = 118) were screened positive for a comorbid eating disorder. Further, 7.0% (*n* = 30) of the participants reported at least one appearance-related suicide attempt (FKS item 18).

The most frequent regions of appearance concerns (FKS item 2) were: skin (62.5%, *n* = 268), nose (44.5%, *n* = 191), hair (41.7%, *n* = 179), breast (25.4%, *n* = 109), mouth (22.4%, *n* = 96), genitals (21.7%, *n* = 93), eyes (21.2%, *n* = 91), muscularity (16.1%, *n* = 69), hands (13.5%, *n* = 58), legs (12.1%, *n* = 58), ears (7.2%, *n* = 31), stomach (6.5%, *n* = 28), buttocks (4.4%, *n* = 19), other facial features (19.8%, *n* = 85), and other regions (20.3%, *n* = 87). The categories “other facial features”, “legs”, “stomach” and “buttocks” were extracted from the open text item “other”.

### Treatment utilization

Of all participants, 62.0% (*n* = 266) reported one or more diagnoses assigned by a professional: depressive disorder including major depression and dysthymia (42.0%, *n* = 180), social anxiety disorder (SAD; 16.8%, *n* = 72), BDD (15.2%, *n* = 65), generalized anxiety disorder (9.1%, *n* = 39), OCD (7.9%, *n* = 34), substance abuse (7.2%, *n* = 31), posttraumatic stress disorder (7.2%, *n* = 31), adjustment disorder (5.4%, *n* = 23), panic disorder (5.1%, *n* = 22), anorexia nervosa (4.4%, *n* = 19), bulimia nervosa (4.0%, *n* = 17), hypochondriasis (3.7%, *n* = 16), specific phobia (3.5%, *n* = 15), skin-picking (3.5%, *n* = 15), personality disorder (3.5%, *n* = 15), other eating disorder (2.8%, *n* = 12), and other mental disorders with each *n* < 10 (8.4%, *n* = 36).

Lifetime rates of treatment utilization and other coping strategies for appearance concerns are presented in Table 2. The most frequent strategies were online research and support by friends and family. Further, research on cosmetic treatments was common, and the rates of cosmetic surgery were comparable to those for mental health treatments. Nevertheless, psychotherapy was the most frequent type of treatment followed by psychopharmacology and general health practitioners. In total, 60.1% (*n* = 258) of the participants reported no lifetime professional mental health treatment for their appearance concerns (i.e. no psychotherapy, psychopharmacotherapy, and/or psychiatric inpatient care) versus 39.9% (*n* = 171) who reported some kind of mental health treatment in the past. Currently, 22.1% (*n* = 95) of the participants were receiving psychopharmacological and/or psychological treatment because of their appearance concerns. Of those, 42.1% (*n* = 40) were diagnosed with BDD.

**Table 2:** Types of treatment and other coping strategies utilized for appearance concerns (*n* = 429)

Treatment utilization	%	(*n*)
Help/treatment sought (lifetime) ^a^		
Psychopharmacotherapy	21.7	(93)
Psychotherapy	29.6	(127)
Psychiatric clinic	12.6	(54)
General practitioner	17.7	(76)
Self-help literature	18.9	(81)
Counselor	5.6	(24)
Healer	8.2	(35)
Research on cosmetic treatments/surgery	63.9	(274)
Cosmetic surgery	25.6	(110)
Friends and family support	49.2	(211)
Research on information (e.g. on the internet)	78.8	(338)
None	3.3	(14)
Other	9.6	(41)
Mental health treatment (current) ^a^		
Psychopharmacotherapy	10.5	(45)
Psychotherapy	17.2	(74)
Cognitive-behavioral therapy	48.6	(36)
Talk therapy	23.0	(17)
Psychodynamic therapy	10.8	(8)
Unknown/other	17.6	(13)

Note. ^a^ Multiple choice items

### Barriers to treatment

The participants endorsed multiple barriers that caused not seeking or delayed seeking of psychological and/or psychopharmacological treatment (see Table 3).

**Table 3:** Treatment barriers (*n* = 429)

Barriers to treatment	%	(*n*)
Stigma, shame, and discrimination barriers		
I felt ashamed of my problems.	49.9	(214)
I was not comfortable discussing my problems with a health professional.	36.6	(157)
I wanted to handle it on my own.	31.2	(134)
I felt ashamed about needing help for my problem.	28.9	(124)
I worried about what people would think if they knew I was in treatment.	21.0	(90)
I would only disclose my appearance concerns, if someone specifically asked about them. ^a^	17.0	(73)
I was afraid of being criticized by my family if I sought psychiatric help.	10.7	(46)
I was scared about being put in a hospital against my will.	6.5	(28)
Treatment satisfaction and perception barriers		
I am unsure if I really need treatment. ^a^	28.9	(124)
Only cosmetic or medical treatments can help with my problems. ^a^	28.2	(121)
I did not think treatment would work.	26.6	(114)
Nobody would understand my problems anyway. ^a^	26.1	(112)
I received treatment before and it did not work.	14.5	(62)
I am not ready for treatment, yet. ^a^	7.9	(34)
I was not satisfied with the services that were available.	7.2	(31)
I do not need treatment. ^a^	3.3	(14)
Logistic and financial barriers		
I was unsure about who to see or where to go.	28.2	(121)
I was worried about how much it would cost.	15.4	(66)
I thought it would be too inconvenient or take too much time.	12.4	(53)
I had problems with transportation or scheduling.	8.9	(38)
I could not choose the provider I wanted to see.	7.7	(33)
I could not get an appointment.	7.7	(33)
Health insurance would not cover treatment.	5.1	(22)

Note. ^a^ Additional items added to the original version of the Barriers to Treatment Questionnaire; Marques et al., 2010 [36])

### Correlates of treatment utilization

First, we compared individuals with lifetime professional mental health help-seeking for their appearance concerns to those without (see Table 4). Treated individuals were older, more often diagnosed with BDD, and reported higher BDD symptom severity as well as more often a likely current major depressive disorder. Those without lifetime mental health treatment reported a higher number of treatment barriers. There were no differences in mental health treatment utilization with respect to gender, current relationship, education, employment, insight, likely eating disorder, other diagnosis, and prior cosmetic surgery. Surprised by the finding that even a former suicide attempt was not associated with receiving any mental health care, we ran additional analyses separately for each type of mental health treatment. Indeed, individuals with prior psychiatric inpatient care reported more often at least one suicide attempt in the past (18.5%, *n* = 10) than individuals who had never been hospitalized did (5.33%, *n* = 20; *Χ*^*2*^(1, 429) = 12.62, *p* < .001, *V* = .17). There was no association of use of psychopharmacological treatment (*p* = .324) or psychotherapy (*p* > .999) with suicide attempt history.

**Table 4:** Comparisons of individuals with and without prior mental health treatment for appearance concerns

	Untreated (*n* = 258)	Treated (*n* = 171)	Comparison	Effect size
	*M* (*SD*) / %	*M* (*SD*) / %	(*p* based on *t*-test/ *Χ*^*2*^-test)	*d / V*
Socio-demographic factors				
Age	27.71 (8.76)	32.51 (10.51)	<.001	0.51
Female	68.6	63.7	.999	0.05
Current relationship	45.3	49.1	.999	0.04
College/university degree	32.6	36.8	.999	0.04
Employed full-time	26.0	35.1	.620	0.10
Clinical factors				
BDD symptom severity	35.80 (8.42)	39.54 (7.80)	<.001	0.46
Insight	3.23 (0.86)	3.33 (0.89)	.999	0.11
Diagnosed with BDD	5.4	29.8	<.001	0.33
Diagnosed with other mental disorder	42.2	53.8	.266	0.11
Eating disorder	29.1	25.1	.999	0.04
Major depressive disorder	47.3	62.6	.027	0.15
Appearance-related suicide attempt	5.0	9.9	.714	0.09
Cosmetic surgery	23.6	28.7	.999	0.06
Number of treatment barriers	4.67 (2.89)	3.73 (3.32)	.027	0.31

Note. *BDD* Body dysmorphic disorder

Second, we modeled the relative effects of the socio-demographic and clinical factors as well as treatment barriers on mental health treatment utilization in a hierarchical logistic regression (s. Table 5). In the first step, higher age increased the likelihood of mental health care use. When entering the clinical factors, age did not remain a significant predictor. Instead, full-time employment turned significant. Additionally, BDD symptom severity and both a prior BDD diagnosis and the diagnosis of another mental disorder increased the likelihood of mental health care utilization. In the final model, these results remained significant. Further, the participants were less likely to report mental health treatment with each treatment barrier they endorsed.

**Table 5:** Predictors of mental health treatment for appearance concerns (adjusted odds ratios with 95% confidence intervals)

	Step 1	Step 2	Step 3	
	OR	OR	OR	95% CI
Socio-demographic factors				
Age (continuous)	1.04**	1.02	1.02	[1.00, 1.05]
Gender (1 = female)	0.81	0.70	0.68	[0.41, 1.11]
Current relationship (1 = yes)	1.07	1.13	1.08	[0.67, 1.72]
College/university degree (1 = yes)	1.06	1.48	1.44	[0.88, 2.37]
Employed full-time (1 = yes)	1.24	1.72*	1.72*	[1.03, 2.90]
Clinical factors				
BDD symptom severity (continuous)		1.07**	1.07**	[1.03, 1.12]
Insight (continuous)		0.74	0.74	[0.53, 1.05]
Diagnosed with BDD (1 = yes)		15.81***	14.76***	[6.80, 32.00]
Diagnosed with other mental disorder (1 = yes)		3.97***	3.93***	[2.29, 6.74]
Eating disorder (1 = yes)		0.78	0.79	[0.47, 1.33]
Major depressive disorder (1 = yes)		1.33	1.39	[0.82, 2.36]
Appearance-related suicide attempt (1 = yes)		1.23	1.32	[0.54, 3.21]
Cosmetic surgery (1 = yes)		1.07	1.01	[0.60, 1.71]
Treatment barriers (continuous)			0.91*	[0.85, 0.99]

Note. *BDD* Body dysmorphic disorder. *** *p* < .001; ** *p* < .01; * *p* < .05

### Correlates of treatment barriers

We computed another series of regressions to examine the relative effects of demographic and clinical features on treatment barriers, both on the overall number of individual barriers as well as on four of the most common barriers: “ashamed of my problems”, “unsure about who to see or where to go”, “unsure if I really need treatment” and “only cosmetic or medical treatments can help with my problems”. Overall, individuals with BDD reported a smaller number of treatment barriers with higher age (*β* = −.12, 95% CI [− 0.07, − 0.01], *p* = .016), a prior BDD diagnosis (*β* = −.17, 95% CI [− 2.38, − 0.59], *p* = .001) and prior cosmetic surgery (*β* = −.10, 95% CI [− 1.36, − 0.02], *p* = .042). Specifically, being diagnosed with BDD (OR = 0.43, 95% CI [0.22, 0.81], *p* = .010) and prior cosmetic surgery (OR = 0.44, 95% CI [0.27, 0.71], *p* = .001) were associated with less shame as a barrier for mental health treatment. Further, a diagnosis of BDD (OR = 0.23, 95% CI [0.10, 0.55], *p* = .001) or another mental disorder (OR = 0.61, 95% CI [0.38, 0.98], *p* = .043) as well as a likely major depressive disorder (OR = 0.49, 95% CI [0.30, 0.82], *p* = .006) were associated with uncertainty about treatment need. A concern about finding an appropriate provider was increased with higher symptom severity (OR = 1.04, 95% CI [1.00, 1.08], *p* = .040) but decreased with prior cosmetic surgery (OR = 0.56, 95% CI [0.33, 0.96], *p* = .034). A preference for cosmetic or medical treatments was strongly positively associated with poorer insight (OR = 2.15, 95% CI [1.43, 3.26], *p* < .001) and prior cosmetic surgery (OR = 2.38, 95% CI [1.43, 3.97], *p* = .001), but decreased with each year of life (OR = 0.97, 95% CI [0.94, 1.00], *p* = .043). The results are displayed in Tables S1 to S5 (see Additional file 1).

## Discussion

The present study aimed to explore previously unstudied aspects of treatment utilization, treatment barriers, and determinants of help-seeking in individuals with BDD in Germany, a country with a longstanding history of universal health care. By using a passive recruitment strategy via an online survey, we conducted, to our knowledge, the largest cross-sectional study on treatment barriers in individuals with BDD to date. We recruited a representative sample of individuals with BDD. The participants reported moderate to severe symptom severity, which is even slightly higher than what is typically found in clinical samples [37–39], relatively poor insight, and typical areas of concern, i.e. skin, nose, and hair (cf. [22]). Of note, common areas of concern found in individuals with eating disorders (e.g. legs, stomach, buttocks) were less frequently reported (4.4 to 12.1%). Thus, individuals in this sample were on average not primarily bothered with weight-related but rather typical BDD concerns. Nonetheless, a third of the participants also presented disordered eating, which matches findings on weight concerns and comorbidity with eating disorders in patients with BDD [40]. We further found a prevalence of 7.3% for appearance-related suicide attempts, which is similar to prior findings in Germany [41] and lies within the range of 1.5–22.2% reported by a recent review on suicidality in BDD [4].

Only 15.2% of all participants had been diagnosed with BDD by a health professional, which is even lower than the finding of 23.3% by Buhlmann [16]. One might argue that there was no improvement in the identification of BDD within the last few years. However, it should be noted that Buhlmann [16] recruited internationally and specifically on BDD-related websites. Moreover, our internet-based self-test might attract more undiagnosed individuals researching their appearance concerns as opposed to already diagnosed BDD patients. It remains unclear, however, whether the reported diagnoses other than BDD (e.g., OCD, SAD) do in fact represent distinct comorbid diagnoses or rather a misdiagnosis of BDD. However, in our sample, only 42.1% of those individuals currently in psychotherapy or psychopharmacological treatment were diagnosed with BDD, although they sought this treatment because of their appearance concerns. If misdiagnosed, the quality of the received treatment is questionable, considering that treating the core symptoms, i.e. specific appearance beliefs and their associated behaviors, is crucial for the treatment to be effective [42, 43].

Results on treatment utilization indicate that most BDD sufferers used concern-related online research rather than help from a health professional or at least professional self-help literature. Further, many individuals seem to be secretive about their disorder – only half of the participants (49.2%) sought support from family or friends. As expected, the minority of individuals with BDD had previously received professional treatment for their appearance concerns. Specifically, about one third (29.6%) had been in psychotherapy and one fourth (21.7%) in psychopharmacological treatment. This is especially remarkable, considering that more than half of the participants (53.4%) also screened positive for a likely current major depressive disorder. Additionally, as participants did not report the specific medication and its dosage, it remains unknown whether individuals received adequate trials, e.g. with SSRIs. Nevertheless, these rates lie within the range of previous findings in international samples with 30.5% of BDD sufferers seeking help from a psychiatrist and 29.5% from a psychologist over their lifetime [17], and 18.6 to 19.8% currently seeking psychosocial or psychopharmacological treatment [16], respectively. Overall, the lifetime utilization of any mental health service in those with current BDD was 39.9% in this study. As expected, only few of the participants indicated financial treatment barriers, most likely because of the extensive health insurance in Germany. Thus, even without major financial barriers, we did not find higher rates of treatment utilization than in the international samples. A recent large population-based study in Germany [44] found that among those with any mental disorder in the past 12 months, 42.6% reported lifetime utilization of any mental health service provider. With respect to Germany, although severely impaired, BDD sufferers use mental health services slightly less frequently than individuals with any mental disorder do and, additionally, often remain undiagnosed.

Several attitudinal treatment barriers were associated with reduced treatment seeking and were highlighted by this study. Most participants reported shame, stigma, and discrimination barriers, which is consistent with prior research on BDD [10, 16, 17, 19] and mental health problems [45]. As shame is associated with avoidance [46], it may directly prevent individuals from disclosing their concerns, e.g. because they fear of being rejected or misunderstood. Further, we found treatment perception to contribute greatly to corresponding utilization: More than one fourth of participants was uncertain whether mental health treatment was needed at all, although they reported significant distress and/or impairment. This may reflect a low perceived need for treatment that was already found as a major treatment barrier for other severe mental disorders in the worldwide WHO World Mental Health Surveys [47]. An underestimation of the perceived need of psychological or psychiatric treatment may be even more relevant for BDD; because of poor insight, individuals often attribute their symptoms solely to their perceived appearance flaws. Our findings underline this with a clear preference for cosmetic or medical treatments or insecurity about the appropriate provider (28.2% each).

As this was a study with a German sample, it was possible to explore which treatment barriers apply to individuals in a country with a stable system of universal and affordable mental health care. In contrast to other countries like the U.S., which has undergone many changes over the years including whether coverage was mandatory as well as its cost for individuals, Germany has mandatory health insurance, which must cover everyone. In 2016, 89% of all German citizens were insured by state health insurance providers [48], which guarantee coverage of psychotherapy and psychopharmacological treatment if indicated. For the remaining citizens who have private health insurance deductibles, co-payments and out-of-pocket expenses, if any, are regulated. Thus, financial barriers were less prominent in this German sample, enabling us to identify crucial attitudinal barriers that influence the decision to seek treatment for individuals with BDD.

We identified several, potentially modifiable factors associated with treatment seeking and barriers to treatment. First, a given BDD diagnosis was clearly associated with treatment utilization and lower levels of treatment barriers, especially shame and perceived need for treatment. In our study, almost one fifth of the participants stated, they would only report their appearance concerns if directly asked for them. Thus, clinicians should routinely screen for BDD especially in patients with prior diagnoses of depression, anxiety disorders, OCD, and substance use disorders [10]. Professionals should further follow diagnostic guidelines [49, 50] and educate patients about their diagnosis and effective (vs. ineffective) treatment options. Second, a special target group for BDD awareness are individuals in cosmetic settings, especially cosmetic surgery. On the one hand, individuals with prior cosmetic surgery perceived less shame as a barrier, possibly due to the fact they already disclosed their concerns explicitly to a health professional. On the other hand, individuals with prior surgery still reported an increased preference for cosmetic procedures and reported to be more certain with their way of help seeking. Therefore, medical professionals should be aware of the fact that they treat a considerable number of patients who could suffer from BDD [9] and thus should use available guidelines for the identification and management of these clients [51, 52]. Third, our data directly reflect that individuals with poorer insight favored cosmetic or medical treatments over mental health treatment. Thus, besides screening procedures, motivational interviewing strategies as described by Wilhelm et al. [42] should ideally be used before the start of a treatment to foster mental health treatment seeking, e.g. in BDD awareness campaigns. Fourth, with respect to demographic characteristics, younger individuals should be of special interest for early interventions, as with increasing age, individuals reported more often the use of mental health treatment and less treatment barriers in general, specifically less reliance on cosmetic or medical treatments. Fifth, individuals tend to seek treatment or perceive their need of treatment more often with increasing symptom burden, i.e. more BDD or depressive symptoms. To encourage sufferers with mild to moderate symptoms to seek help, mental health literacy campaigns and stepped care approaches should be fostered. Online research was the most frequent coping strategy used, but comparably few individuals used self-help books. Guided self-management of symptoms, e.g. via online delivered psychotherapy or smartphones, could bridge this gap, addresses many of the logistical and shame related barriers we identified and has already shown promising effects in BDD [53–55].

While interpreting the current results, some limitations have to be considered. Due to its cross-sectional nature, we cannot make any assumptions regarding causality of our findings. Because of the online setting, the participants were not diagnosed by trained professionals. Thus, it cannot be ruled out that reported appearance concerns, disordered eating, and depressive symptoms are better explained by other mental disorders, substance use or a medical condition. Most importantly, no participant could be excluded from the BDD group because of visible appearance flaws or rather having an eating disorder than BDD (although participants specifically indicated that their appearance concerns were not primarily weight-related). In addition, the utilized German self-report measure for BDD, although similar to common screening instruments in English (e.g. Body Dysmorphic Disorder Questionnaire, [56]) and validated by the cut-off of the FKS, have not been validated thoroughly, yet. Because of the self-selected sample, the results have limited generalizability. For example, one may argue that individuals taking an online self-test on the website of a psychotherapy clinic are presumably motivated to seek information on problems they are already aware of. Thus, participants show at least some insight, and may already consider psychotherapeutic treatment or may be particularly treatment experienced. Yet, the results show that this is a sample with a particularly low rate of lifetime mental health treatment. The actual rates of treatment utilization may thus be even lower than indicated by this sample. Given the large proportion of individuals without mental health treatment in the past, comparisons between treated and untreated BDD sufferers were possible. Although the sample size was large, post hoc power analyses indicated that it was not sufficient to detect small effect sizes for these group comparisons reliably. Hence, larger sample sizes are needed in future studies. As a strength of the online sampling, this study is independent of relevant barriers related to face-to-face contact and thus especially ecological valid including insights about individuals who could not have been included otherwise. Further, due to the selected sample, our screening procedure for BDD may work even better than in the general population. As discussed above, the clinical characteristics of this sample revealed a clinically significant impaired sample and proved to be similar to clinical samples. The self-test continuously attracted users and had a low rate of dropouts. Researchers should expand on the development of such educational programs with the possibility of feedback, and evaluate their effects on help-seeking behaviors in future studies.

## Conclusions

In sum, we demonstrated that the diagnostic and treatment status in BDD is still unsatisfactory. Although BDD is a severe, but treatable condition, only a small group of BDD sufferers is adequately diagnosed and affiliated with the mental health care system even in a country with extensive coverage of psychotherapy by the health system. We appeal to further raise awareness for BDD in possible sufferers, patients and healthcare professionals, including psychotherapists and psychiatrists. Special target groups are individuals in cosmetic and medical settings presenting for strong body dissatisfaction and firmly held appearance beliefs. Also, younger individuals and mild to moderately burdened sufferers should be addressed more often. It is crucial to provide individuals with BDD with easily accessible information regarding the diagnosis and effective treatment strategies. Our self-test could be a relatively easy first step to overcome assessment and treatment barriers. In fact, it might be a first step in a stepped care model that assesses patients’ symptom severity (and ultimately other factors relevant to the personalization of treatment as well) and then guides them to psychoeducational websites, prevention programs, online CBT, smartphone treatment apps, or specialized face-to-face treatments.

## Supplementary information

**Additional file 1.** This pdf-file includes the results of the regression analyses not reported in detail in the results section. Table S1 Predictors of number of treatment barriers. Table S2 Predictors of treatment barrier “I felt ashamed of my problems”. Table S3 Predictors of treatment barrier “Only cosmetic or medical treatments can help with my problems”. Table S4 Predictors of treatment barrier “I am unsure if I really need treatment”. Table S5 Predictors of treatment barrier “I was unsure about who to see or where to go”.

## Data Availability

The data that support the findings of this study are available on request from the corresponding author [UB]. The data are not publicly available due to them containing information that could compromise research participant consent.

## Abbreviations

BDD: Body dysmorphic disorder
BTQ: Barriers to Treatment Questionnaire
DSM-5: 5th edition of the Diagnostic and Statistical Manual of Mental Disorders
FKS: Body Dysmorphic Symptoms Inventory
OCD: Obsessive-compulsive disorder
PHQ-9: Patient’s Health Questionnaire depression scale
SAD: Social anxiety disorder
SSRIs: Selective serotonin-reuptake inhibitors
