# Supplementary material for: Treatment utilization and treatment barriers in individuals with body dysmorphic disorder
Source: BMC Psychiatry. 2020 Feb 18;20:69. doi: 10.1186/s12888-020-02489-0 (PMC7027080; doi:10.1186/s12888-020-02489-0)
Supplement: Supplementary file 1 — Additional file 1. This pdf-file includes the results of the regression analyses not reported in detail in the results section. Table S1 Predictors of number of treatment barriers. Table S2 Predictors of treatment barrier “I felt ashamed of my problems”. Table S3 Predictors of treatment barrier “Only cosmetic or medical treatments can help with my problems”. Table S4 Predictors of treatment barrier “I am unsure if I really need treatment”. Table S5 Predictors of treatment barrier “I was unsure about who to see or where to go”. [file 12888_2020_2489_MOESM1_ESM.pdf]

Table S1

Predictors of number of treatment barriers (standardized beta with 95% confidence intervals)

	Step 1	Step 2	
	β	*β*	95% CI
Socio-demographic factors			
Age (continuous)	-0.17**	-0.12*	[-0.07, -0.01]
Gender (1 = female)	-0.05	-0.06	[-1.02, 0.23]
Current relationship (1 = yes)	-0.06	-0.06	[-0.98, 0.19]
College/university degree (1 = yes)	-0.07	-0.06	[-0.99, 0.25]
Employed full-time (1 = yes)	-0.01	-0.01	[-0.73, 0.57]
Clinical factors			
BDD symptom severity (continuous)		0.09	[-0.02, 0.08]
Insight (continuous)		0.02	[-0.35, 0.50]
Diagnosed with BDD (1 = yes)		-0.17**	[-2.38, -0.59]
Diagnosed with other mental disorder (1 = yes)		-0.08	[-1.11, 0.17]
Eating disorder (1 = yes)		0.04	[-0.37, 0.93]
Major depressive disorder (1 = yes)		0.08	[-0.18, 1.14]
Appearance-related suicide attempt (1 = yes)		0.06	[-0.50, 1.85]
Cosmetic surgery (1 = yes)		-0.10*	[-1.36, -0.02]

Note. BDD = Body dysmorphic disorder. *** $p < .001$. ** $p < .01$. * $p < .05$.

Table S2

Predictors of treatment barrier "I felt ashamed of my problems" (adjusted odds ratios with 95% confidence intervals)

	Step 1	Step 2	
	OR	OR	95% CI
Socio-demographic factors			
Age (continuous)	0.98	0.99	[0.96, 1.01]
Gender (1 = female)	1.02	1.03	[0.67, 1.60]
Current relationship (1 = yes)	0.94	0.87	[0.58, 1.31]
College/university degree (1 = yes)	1.24	1.23	[0.80, 1.90]
Employed full-time (1 = yes)	0.72	0.71	[0.45, 1.13]
Clinical factors			
BDD symptom severity (continuous)		1.03	[0.99, 1.07]
Insight (continuous)		0.92	[0.69, 1.23]
Diagnosed with BDD (1 = yes)		0.43*	[0.22, 0.81]
Diagnosed with other mental disorder (1 = yes)		1.05	[0.68, 1.64]
Eating disorder (1 = yes)		0.97	[0.62, 1.52]
Major depressive disorder (1 = yes)		1.15	[0.73, 1.82]
Appearance-related suicide attempt (1 = yes)		0.82	[0.36, 1.87]
Cosmetic surgery (1 = yes)		0.44**	[0.27, 0.71]

Note. BDD = Body dysmorphic disorder. *** $p < .001$. ** $p < .01$. * $p < .05$.

Table S3

Predictors of treatment barrier "Only cosmetic or medical treatments can help with my problems" (adjusted odds ratios with 95% confidence intervals)

	Step 1	Step 2	
	OR	OR	95% CI
Socio-demographic factors			
Age (continuous)	0.97*	0.97*	[0.94, 1.00]
Gender (1 = female)	1.28	1.16	[0.69, 1.93]
Current relationship (1 = yes)	0.83	0.87	[0.54, 1.40]
College/university degree (1 = yes)	0.72	0.89	[0.53, 1.47]
Employed full-time (1 = yes)	1.11	1.18	[0.70, 2.00]
Clinical factors			
BDD symptom severity (continuous)		1.01	[0.97, 1.05]
Insight (continuous)		2.15***	[1.43, 3.26]
Diagnosed with BDD (1 = yes)		0.94	[0.47, 1.88]
Diagnosed with other mental disorder (1 = yes)		0.90	[0.54, 1.50]
Eating disorder (1 = yes)		1.05	[0.63, 1.74]
Major depressive disorder (1 = yes)		1.40	[0.83, 2.37]
Appearance-related suicide attempt (1 = yes)		1.51	[0.65, 3.53]
Cosmetic surgery (1 = yes)		2.38**	[1.43, 3.97]

Note. BDD = Body dysmorphic disorder. *** $p < .001$. ** $p < .01$. * $p < .05$.

Table S4

Predictors of treatment barrier "I am unsure if I really need treatment" (adjusted odds ratios with 95% confidence intervals)

	Step 1	Step 2	
	OR	OR	95% CI
Socio-demographic factors			
Age (continuous)	0.97*	0.98	[0.96, 1.01]
Gender (1 = female)	0.89	0.88	[0.54, 1.43]
Current relationship (1 = yes)	0.86	0.80	[0.50, 1.27]
College/university degree (1 = yes)	1.17	.92	[0.57, 1.49]
Employed full-time (1 = yes)	1.22	1.08	[0.65, 1.81]
Clinical factors			
BDD symptom severity (continuous)		0.99	[0.95, 1.03]
Insight (continuous)		0.89	[0.65, 1.23]
Diagnosed with BDD (1 = yes)		0.23**	[0.10, 0.55]
Diagnosed with other mental disorder (1 = yes)		0.61*	[0.38, 0.98]
Eating disorder (1 = yes)		1.34	[0.81, 2.22]
Major depressive disorder (1 = yes)		0.49*	[0.30, 0.82]
Appearance-related suicide attempt (1 = yes)		0.23	[0.05, 1.06]
Cosmetic surgery (1 = yes)		0.82	[0.48, 1.41]

Note. BDD = Body dysmorphic disorder. *** $p < .001$. ** $p < .01$. * $p < .05$.

Table S5

Predictors of treatment barrier "I was unsure about who to see or where to go" (adjusted odds ratios with 95% confidence intervals)

	Step 1	Step 2	
	OR	OR	95% CI
Socio-demographic factors			
Age (continuous)	0.98	0.99	[0.96, 1.01]
Gender (1 = female)	1.04	1.09	[0.68, 1.77]
Current relationship (1 = yes)	1.06	1.00	[0.64, 1.57]
College/university degree (1 = yes)	0.66	0.67	[0.41, 1.09]
Employed full-time (1 = yes)	1.16	1.20	[0.73, 1.98]
Clinical factors			
BDD symptom severity (continuous)		1.04*	[1.00, 1.08]
Insight (continuous)		0.80	[0.58, 1.11]
Diagnosed with BDD (1 = yes)		0.67	[0.33, 1.34]
Diagnosed with other mental disorder (1 = yes)		0.85	[0.53, 1.38]
Eating disorder (1 = yes)		0.74	[0.45, 1.23]
Major depressive disorder (1 = yes)		0.93	[0.57, 1.53]
Appearance-related suicide attempt (1 = yes)		1.19	[0.51, 2.76]
Cosmetic surgery (1 = yes)		0.56*	[0.33, 0.96]

Note. BDD = Body dysmorphic disorder. *** $p < .001$. ** $p < .01$. * $p < .05$.
